# Relationship between radiographic changes and symptoms or physical examination findings in subjects with symptomatic medial knee osteoarthritis: a three-year prospective study

**DOI:** 10.1186/1471-2474-11-269

**Published:** 2010-11-24

**Authors:** Naoshi Fukui, Shoji Yamane, Satoru Ishida, Konagi Tanaka, Riako Masuda, Nobuho Tanaka, Yozo Katsuragawa, Sakiko Fukui

**Affiliations:** 1 Clinical Research Center, National Hospital Organization Sagamihara Hospital, Sakuradai 18-1, Minami-ku, Kanagawa 252-0315, Japan; 2 Department of Orthopaedic Surgery, International Medical Center of Japan, Toyama 1-21-1, Shinjuku-ku, Tokyo 162-8655, Japan; 3 Graduate School of Nursing, Japanese Red Cross College of Nursing, Hiro-o 4-1-3, Shibuya-ku, Tokyo 150-0012, Japan

## Abstract

**Background:**

Although osteoarthritis (OA) of the knee joints is the most common and debilitating joint disease in developed countries, the factors that determine the severity of symptoms are not yet understood well. Subjects with symptomatic medial knee OA were followed up prospectively to explore the relationship between radiographic changes and symptoms or physical examination findings.

**Methods:**

One-hundred six OA knees in 68 subjects (mean age 71.1 years; 85% women) were followed up at 6-month intervals over 36 months. At each visit, knee radiographs were obtained, symptoms were assessed by a validated questionnaire, and the result of physical examination was recorded systematically using a specific chart. Correlations between the change of radiographs and clinical data were investigated in a longitudinal manner.

**Results:**

During the study period, the narrowing of joint space width (JSW) was observed in 34 joints (32%). Although those knees were clinically or radiographically indistinguishable at baseline from those without JSW narrowing, differences became apparent at later visits during the follow-up. The subjects with knees that underwent JSW narrowing had severer symptoms, and the symptoms tended to be worse for those with higher rates of narrowing. A significant correlation was not found between the severity of symptoms and the growth of osteophytes. For the knees that did not undergo radiographic progression, the range of motion improved during the follow-up period, possibly due to the reduction of knee pain. Such improvement was not observed with the knees that underwent JSW narrowing or osteophyte growth.

**Conclusion:**

The result of this study indicates that the symptoms of knee OA patients tend to be worse when JSW narrowing is underway. This finding may explain, at least partly, a known dissociation between the radiographic stage of OA and the severity of symptoms.

## Background

Osteoarthritis is a common, age-related disorder of the synovial joints, which primarily involves articular cartilage, synovium, and subchondral bones. With increasing longevity, OA has become the most prevalent form of joint disease in developed countries [1]. Knee OA is particularly important in view of its prevalence and association with disability [2,3], which makes this disease a large economic and medical burden to society [1,4].

Pathologically, OA is characterized by focal loss of articular cartilage in weight-bearing areas and new bone formation at joint margins. With the progression of the disease, these changes become apparent on plain radiographs [5-7]. The extent of cartilage loss can be estimated by measuring joint space width (JSW) on radiographs obtained in weight-bearing positions. Newly formed bone tissue is noted as osteophytes at joint margins.

Knee OA patients most often complained of joint pain, stiffness, restriction of joint motion, and cracking or crepitus within the joints [8]. Among these complaints, joint pain is particularly important because it largely accounts for patients' disability with the disease [3,9,10]. These clinical problems are supposed to arise in association with the above-mentioned pathological changes. However, the severity of a patient's symptoms often does not correlate to the degree of the disease progression evaluated on radiographs [11,12]. In clinics, patients in the early stages of knee OA often have severe knee pain and disability, while those in advanced stages may have only minor symptoms [11,13-17]. Thus, one can not simply assume that the degree of radiographic progression determines the severity of symptoms in knee OA patients.

Knee OA is a highly heterogeneous disease in terms of progression. Previous studies have shown that some OA knees remain stable for years, while others undergo rapid progression [11-13,16,18-21]. Considering this heterogeneity, it may be possible that the patients undergoing disease progression could be clinically distinguishable from those in a stable condition. However, currently it is not known whether the symptoms or physical findings are indeed related to the progression of radiographic changes in knee OA subjects.

To clarify this, we conducted a follow-up study of the subjects with symptomatic knee OA, and investigated the relationship between radiographic progression and symptoms or physical examination findings. The study has revealed several novel aspects in their correlation.

## Methods

### Subjects

Subjects for this study were recruited at a community medical center from among the patients seeking medical care for symptomatic knee OA. The study was performed under the approval of the institutional review board, and informed consent was obtained in writing from each subject. To be included in the study, the subject had to be 50 years of age or older, in good general health, and have primary knee OA with medial involvement at least in one knee. The persons who had significant impairment in the spine or lower extremities were not requested to participate. The diagnosis of primary knee OA was based on the criteria determined by the American Rheumatism Association with some modifications [8]. That is, the patient had to have persistent knee pain for 3 months or more, and had to have at least one definite osteophyte visible on their radiographs. The involvement of the medial compartment was determined radiographically by the narrowing of the joint space or the presence of a marginal osteophyte in that compartment, with the help of radiographic atlases of knee OA [6,7]. The presence of OA changes in the patellofemoral compartment was not an exclusion criterion, but knees with three-compartmental involvement were not included in the study. A history of a previous injury or surgery was another exclusion criterion. In this investigation, we planned to monitor the progression of the disease primarily by the narrowing of the JSW. For this reason, knees in which the joint space was already obliterated were not eligible for the study. Thus, the inclusion of respective knee joints in the study was finally determined by the radiographs at the enrollment, as described later.

At enrollment, the age, sex, and body mass index (BMI) of the subjects were recorded, and standard blood tests were conducted to determine the serum concentration of C-reactive protein (CRP), erythrocyte sedimentation rate (ESR), and the level of rheumatoid factor. At the enrollment and every 6 months thereafter, radiographs were obtained, and clinical assessment and physical examination were performed repeatedly until the final follow-up at 36 months.

During the study period, all subjects were treated conservatively, although two of them failed to be managed and underwent surgery, as described later. Conservative treatment was started from non-pharmacological therapy that consisted of patient education, muscle strengthening exercise, range of motion exercise, and weight loss when indicated. If the symptoms did not improve, an ointment or patches containing non-steroidal anti-inflammatory drugs (NSAIDs) were prescribed for the subjects. NSAIDs might be given orally to those with severe symptoms. Hyaluronate was administered intra-articularly when the symptoms were intolerable, but corticosteroid was not given to any subjects in this series.

### Radiography

At each visit, three radiographs were obtained on each evaluated knee. An anteroposterior (AP) view was obtained in the standing position with the knee in full extension. An axial view was obtained in a 45 degree-flexed position with the subject supine on an X-ray table, following the method of Merchant *et al*. [22] Posteroanterior (PA) radiographs were obtained in the weight-bearing fixed-flexion position with the feet externally rotated 10° and the toes, knees and thighs touching the wall on which the film cassette was placed [23,24]. Before radiography, the outline of the subject's feet was traced on a paper sheet taped to the floor for repositioning the limb in case of repeated exposures. Immediately after the acquisition, parallel alignment of the joint and the x-ray beam was confirmed on each radiograph. When the alignment was poor, the radiograph was taken again after adjusting the tube angle and position. In the PA radiograph, in particular, the alignment was examined with care: for this radiograph to be acceptable, the tibial spines should be located beneath the femoral notch, and the distance between the anterior and posterior margins of the medial tibial plateau should be equal to or less than 1.5 mm [25]. For the reason mentioned earlier, knee joints whose medial joint space was already closed on the PA radiograph at the enrollment were not included in this study.

### Evaluation of radiographs

Progression of OA was determined radiographically by the progression of JSW narrowing and the change in the severity of osteophytosis. In order to evaluate the change of JSW, PA radiographs were converted to digitized images using a laser film digitizer (LD-5500, Konica Minolta MG, Tokyo, Japan), which can scan films at a maximum resolution of a 50-μm focal spot with 256 levels of gray. On these images, JSW was defined as the minimum distance between the femur and tibia in the medial femorotibial compartment. The JSW was measured on the computer system under a proper magnification, which was corrected for magnification by the image of a magnification marker (a steel ball 11 mm in diameter) that was affixed to the lateral aspect of the knee before the acquisition of radiographs.

Severity of osteophytosis was evaluated by the total of severity scores determined at respective sites of the joint on AP and axial view radiographs. On the AP radiograph, formation of osteophytes was rated at the four sites in lateral and medial aspects of the femur and tibia, respectively, using a scale of 0 (absent) to 3 (severest), referring to the standardized radiographic atlases [6,7]. On the axial view radiograph, osteophytes were rated in the same manner at the two sites in lateral and medial aspects of the patellofemoral compartment, referring to the atlas [7]. Thus, the severity of osteophytosis was determined for each knee by the summation of those scores which ranged from 0 (absent) to 18 (severest). These scores were assigned independently by two experienced readers (NF and KT) who were blinded to patient identity or chronological orders. When the score was discordant between them, a third independent reader (YK) made the adjudication on the score in a blinded manner. Inter-reader agreement of the first two readers in the rating was κ = 0.62 (p < 0.001).

### Clinical assessment

Symptoms of the patients were evaluated by the Japanese Knee Osteoarthritis Measure (JKOM), a questionnaire designed to evaluate symptoms and functional disabilities with knee OA in the Japanese cultural lifestyle [26]. This is a self-completed questionnaire that consists of a visual analogue scale (VAS) for the degree of global knee pain, and 25 items covering the following four categories: 8 items for pain and stiffness, 10 for conditions in daily life, 9 for general activities, and 2 for health conditions. The overall result was assessed using the result of VAS and the sum of the scores for these 25 items, which ranged from 25 (no complaint) to 133 (possible severest condition).

Physical examination was performed systematically using a specific chart. In the examination, the presence of local warmth in the medial joint space, swelling, tenderness on the medial joint line, crepitation, and range of motion of the joint were examined and recorded. ROM was measured in an assisted-active manner. For this, the subjects were requested to lie supine, and extend or flex each knee as far as possible until discomfort, with the assistance of an examiner, if needed. The knee extension angle and flexion angle were measured and recorded in degrees, respectively, using a large standard goniometer.

### Statistical analysis

Statistical significances were determined by Fisher's exact test, and paired or unpaired *t*-test. The relationship between the JKOM score and radiographic progression was analyzed by mixed model analysis, in which the JKOM score and the occurrence of radiographic progression (progression of JSW narrowing or increase in osteophyte score) were included as fixed effects, whereas the follow-up period was entered as a random effect. Receiver operating characteristic (ROC) analysis was employed to determine the predictability of the JKOM score for the occurrence of radiographic progression. The level of significance was set at P = 0.05. All analyses were carried out using the SAS statistical software for Windows, version 9.1 (SAS Institute, Cary, NC, USA).

## Results

Among the 84 subjects enrolled in the study, 68 completed the 36-months follow-up. There were 10 males and 58 females, with a mean age of 71.1 ± 8.4 and a mean BMI of 25.5 ± 2.5. There were no significant difference in any of the demographic or clinical characteristics between the subjects who were fully followed up and those lost to follow-up (data not shown). In the 68 subjects who completed the follow-up, 30 knees in 30 subjects were not eligible for the study, and the evaluation was performed on the remaining 106 knees. The reasons for the exclusion of the 30 knees were as follows: medial compartment was not the primary site of involvement (14 knees), obliteration of the medial joint space on a PA radiograph (9 knees), tricompartmental involvement (5 knees), history of previous knee surgeries (2 knees). During the follow-up period, prosthetic surgery was performed on 2 knees at 28 and 31 months after the enrollment, respectively. For those joints, the data prior to the surgery were included in the analyses.

**Figure 1:**
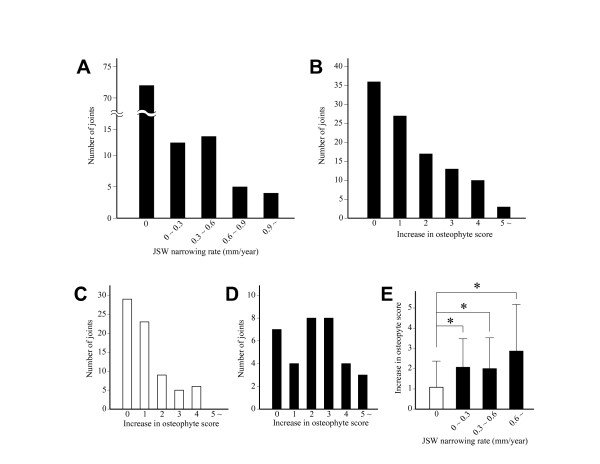
**Change of JSW and osteophyte score during the follow-up period**. A and B. Distribution of the JSW narrowing rate (A) and increase in osteophyte score (B) among evaluated knee joints. C and D. Distribution of the increase of osteophyte score among non-progressed (C) and progressed joints (D). E. Increase in osteophyte score relative to JSW narrowing rate. Results are shown by mean + SD. *, P < 0.05, unpaired *t*-test. In C-E, open and solid bars indicate non-progressed and progressed joints, respectively.

The average rate of JSW narrowing for those 106 joints was 0.13 ± 0.14 mm/year. The change of JSW differed considerably among the joints. During the study period, reduction of JSW was observed in 32% of the joints (34 knees), while narrowing was not detected in the remaining 68% (72 knees) (Figure 1A). Thus, the average rate of narrowing calculated only for the former joints was as high as 0.46 ± 0.38 mm/year. Considering this difference in JSW narrowing among joints, in the following analyses, results were often compared between the knees that underwent JSW narrowing (progressed joints) and those that evaded narrowing (non-progressed joints). None of the baseline characteristics we evaluated differed significantly between these two groups of subjects (Table 1).

**Table 1:** Baseline characteristics of the subjects by radiographic progression

	**Subjects with progressed joints**^**a**^	**Subjects with non-progressed joints**^**b**^	**p value**^**c**^
Number of subjects	26 (34 joints)	42 (72 joints)	
Male	3 (4 joints)	7 (14 joints)	0.730 (0.413)^d^
Female	23 (30 joints)	35 (58 joints)	
Age	70.6 ± 9.4	71.7 ± 6.5	0.606
BMI	25.7 ± 2.8	25.2 ± 2.2	0.883
JKOM (total score)	63.6 ± 16.2	58.7 ± 15.1	0.389
K-L score	1.84 ± 0.64	1.85 ± 0.67	0.937
JSW (mm)	2.86 ± 1.18	3.18 ± 1.21	0.411
Osteophyte score	3.96 ± 2.18	3.71 ± 2.21	0.827

^a^subjects who had at least one progressed joint; ^b^subejcts without progressed joints; ^c^determined by Fischer's exact test; ^d^sex ratio of subjects (joints). BMI: body mass index; JKOM: Japanese Knee Osteoarthritis Measure (reference 26); JSW: joint space width; K-L score: Kellgren-Lawrence score (reference 5).

In our series of OA knees, osteophyte growth occurred more often than JSW narrowing. During the study period, the osteophyte score increased in 66% of the knees (Figure 1B), which was almost double the number of knees that underwent JSW narrowing. There was some discordance between the increase in the osteophyte score and the progression of JSW narrowing. Although those changes often occurred together, the osteophyte score increased in 54% of the non-progressed joints (Figure 1C), while it remained virtually unchanged in 21% of the progressed joints (Figure 1D). Among the progressed joints, the increase of the osteophyte score tended to be greater in the knees with higher rates of narrowing, though it did not reach the level of statistical significance (Figure 1E).

**Figure 2:**
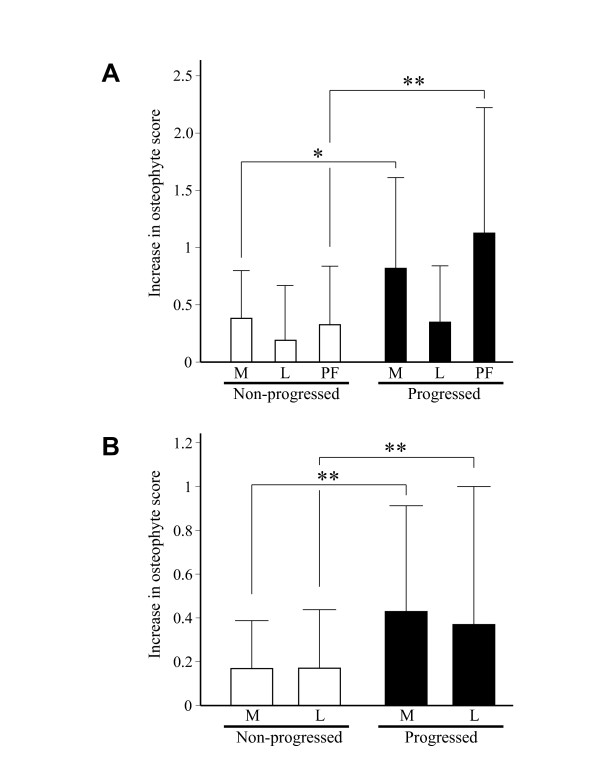
**Change of osteophyte score at respective compartments within knee joint**. A. Increase of osteophyte score in medial (M), lateral (L), and patellofemoral (PF) compartments in non-progressed and progressed joints. B. Increase of osteophyte score in medial (M) and lateral aspects (L) of patellofemoral compartment in non-progressed and progressed joints. Results are shown by mean + SD. *, P < 0.05, and **, P < 0.01, unpaired *t*-test.

We next compared growth of the osteophytes among the three compartments within the knee joint. In progressed joints, significant increase of the osteophyte score was observed not only in the medial compartment but also in the patellofemoral compartments (Figure 2A). In the patellofemoral compartment, the score increased equally on the lateral and medial aspects (Figure 2B), implying that osteophyte growth in that compartment could be independent from OA changes in the medial compartment.

**Figure 3:**
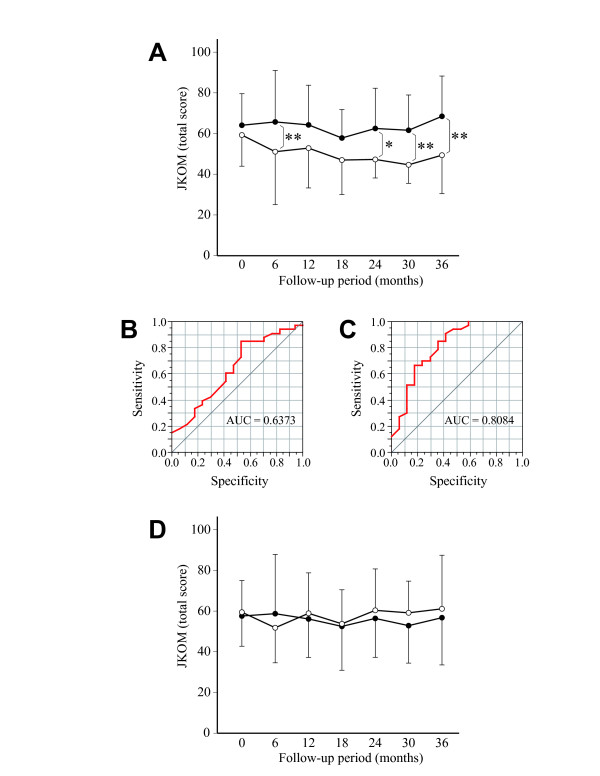
**Relationship between symptoms and radiographic changes**. A. Longitudinal change of JKOM score in the subjects who had at least one progressed joint (closed circle) and those without progressed joints (open circle). **, P < 0.01, unpaired *t*-test. B and C. Results of ROC analysis of JKOM score for the prediction of JSW narrowing at enrollment (B) and at 30 months (C), resepctively. AUC, area under the curve. D. JKOM score in subjects whose osteophyte score increased 2 or more in at least one knee (closed circle) and that in subjects whose increase in score was less than 2 in either knee (open circle) at baseline and every 6 months. In A and D, higher JKOM score indicates severer symptoms. Results are shown by mean + or - SD.

In the following analysis, the relationship between the symptoms and radiographic changes was investigated. First, the JKOM score was compared between the subjects who underwent JSW narrowing and those without narrowing. At baseline, the JKOM score was similar for those two groups of subjects (Figure 3A). At later visits, the score for the subjects with non-progressed joints declined gradually over time, while that for the subjects with progressed joints remained high until the final visit. Thus, the difference in the score between these groups of subjects was significant at 6, 24, 30, and 36 months, respectively. As the JKOM score differed significantly between the groups at those time points, we next performed ROC analysis and evaluated the predictability of that score for the progression of JSN narrowing. The result of this analysis indicated that the prognostic value of the score as expressed by the area under the curve (AUC) was lowest at enrollment (0.6373) (Figure 3B), and highest at 30 months (0.8084) (Figure 3C), followed by that at 30 months (0.7986) and 36 months (0.7674).

Next, the JKOM score was analyzed against the change in the osteophyte score. In this analysis, the score was compared between subjects whose osteophyte score increased by 2 or more in at least one knee, and those whose increase was less than 2 in both knees, considering the distribution of the score (Figure 1B). Unlike the former result, the JKOM score did not change significantly between these two groups of subjects throughout the study period (Figure 3D).

**Figure 4:**
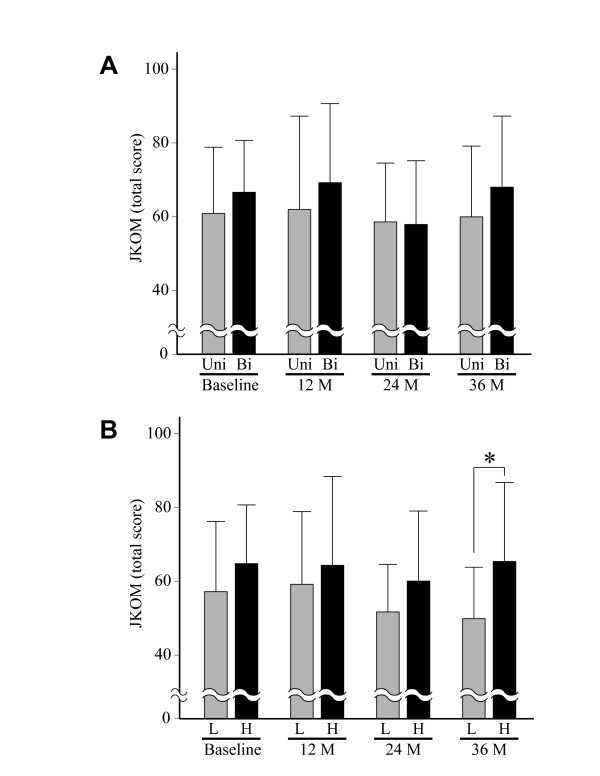
**Relationship between symptoms and change of JSW in subjects who had at least one progressed joint**. A. JKOM score of the subjects who had progressed joints unilaterally (Uni) was compared with that of the subjects who had such joints bilaterally (Bi) at baseline and every 12 months. B. JKOM score of the subjects who had lower rates of JSW narrowing (<0.32 mm/year) (L) was compared with that of the subjects with higher rates of narrowing (≥0.32 mm/year) (H) at baseline and every 12 months. Higher JKOM score indicates severer symptoms. Results are mean + SD. *, P < 0.05, unpaired *t*-test.

Based upon these findings, we further investigated the relationship between JSW narrowing and symptoms in the subjects who had at least one progressed joint. First, the JKOM score was compared between the subjects who had two (bilateral) progressed joints and those with only one. Contrary to our expectation, the JKOM score did not change significantly between those two groups of subjects (Figure 4A). Next, we compared the symptoms regarding the rate of JSW narrowing. For this, subjects with at least one progressed joint were divided into two groups, those for whom the rate of JSW narrowing was equal to or above 0.32 mm/year, and those who experienced less, based on the median rate of narrowing for the progressed joints. A subject who had two progressed joints was categorized by the greater rate of the two. This analysis showed that the JKOM score tended to be higher for subjects with the greater rates of JSW narrowing, though a significant difference was found only at the final visit at 36 months (Figure 4B).

**Figure 5:**
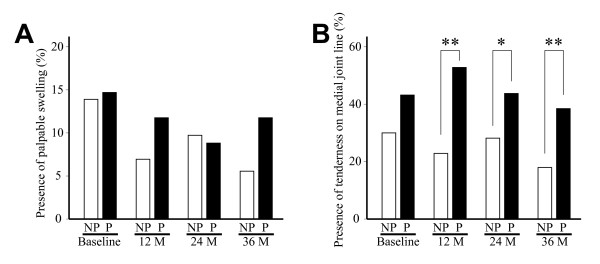
**Relationship between physical examination findings and JSW narrowing**. Presence of palpable swelling (A) and tenderness on medial joint line (B) was compared between non-progressed (NP) and progressed joints (P) at baseline and every 12 months. Open and solid bars indicate progressed and non-progressed joints, respectively. *, P < 0.05, and **, P < 0.01, unpaired *t*-test.

We also investigated the relationship between the result of physical examination and radiographic progression. First, the frequency of joint swelling was compared between the progressed and non-progressed joint at baseline and at each following 12-month interval. Although knee swelling tended to be more often present in the progressed joints, the difference was not significant throughout the study period (Figure 5A). Next, the frequency of tenderness on the medial joint line was compared between those groups. Although not significant at baseline, the frequency of tenderness was significantly higher for the progressed joints at 12, 24, and 36 months, respectively (Figure 5B).

**Figure 6:**
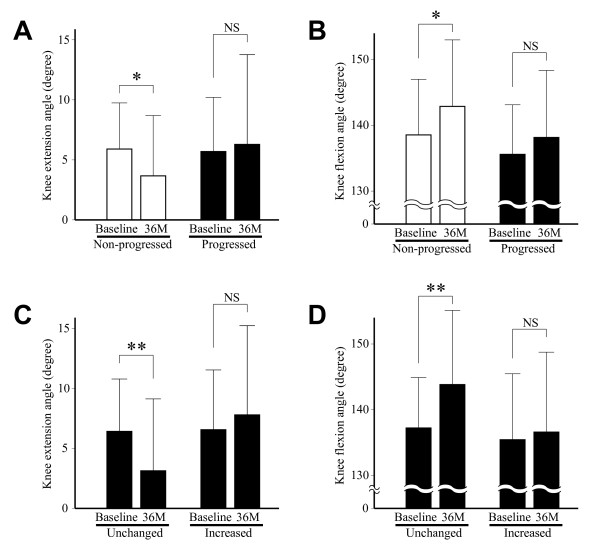
**Relationship between radiographic progression and change of knee extension and flexion angles**. A and B. Knee extension angle (A) and flexion angle (B) at baseline and final visit after 36 months (36M) are shown for non-progressed and progressed joints, respectively. Open and solid bars indicate progressed and non-progressed joints, respectively. C and D. Knee extension angle (C) and flexion angle (D) at baseline and final visit after 36 months (36M) are shown for the joints with little increase in osteophyte score (0 or 1) (Unchanged) and those with substantial increase in the score (2 or more) (Increased), respectively. *, P < 0.05, and **, P < 0.01, paired *t*-test.

The change of restriction in joint motion over the study period was analyzed against the progression of JSW narrowing or the increase in osteophyte score. First, the change of knee extension angle was compared between the progressed and non-progressed joints. For the non-progressed joints, the extension angle improved significantly between the baseline and the final visit, but such improvement was not observed with the progressed joints (Figure 6A). Similarly, the knee flexion angle improved significantly with the non-progressed joints, but that trend was not seen with the progressed joints (Figure 6B). Next, the change of flexion or extension angle was compared between the knees with little increase in osteophyte score (0 or 1) and those with substantial increase (2 or more). The result showed that both of those angles improved significantly for the former knees, while such improvement was not observed with the latter knees (Figure 6C and 6D).

## Discussion

Progression of knee OA is most often evaluated by the narrowing of JSW on weight-bearing radiographs. To date, many studies have reported the change of JSW in knee OA [27-30]. Although there is some disagreement in the rate of JSW narrowing among those studies, a recent meta-analysis estimated that the average rate of JSW narrowing could be 0.13 ± 0.15 mm/year [31]. This rate of narrowing and standard deviation are very close to our current result, supporting the validity of our methods of subject selection and radiographic measurement.

Besides the change of JSW, formation of osteophytes is another radiographic feature for the progression of OA. The result of this study revealed that these changes may not occur in parallel in OA knees. For instance, the osteophyte score increased in only half of the non-progressed joints (Figure 1C). Such dissociation between JSW narrowing and osteophyte growth might be related to the difference in the mechanisms for the respective changes. For example, transforming growth factor (TGF)-β1 is currently considered to be responsible for the formation of osteophytes [32,33]. However, this protein has been shown to have protective effects on cartilage [34,35]. Thus, if TGF-β1 is abundantly expressed within OA joints, osteophytes may develop without the loss of cartilage matrix. A previous study has shown that JSW narrowing and osteophyte growth have their own risk factors [36], which also suggests the difference in the mechanisms for those changes.

In the next analysis, we found a significant difference in the severity of symptoms between the subjects who underwent JSN narrowing and those who did not (Figure 3A). Consistently, a significant difference was observed with the presence of tenderness on the medial joint line between the progressed joints and non-progressed joints (Figure 5B). These trends were not clear at baseline, but became evident at later visits. In understanding these results, it may be noted that all subjects in this study were those who were referred to our clinics for their knee symptoms. We think that if subjects had been recruited from the general population by radiographic screening and asymptomatic subjects had been included, the relationship between the symptoms and JSW narrowing could have been clearer.

Although no previous studies have investigated the relationship between radiographic changes and symptoms of knee OA in a longitudinal manner as we did in this work, several investigators have reported that knee pain at baseline is a risk factor for the progression of JSW narrowing [36-39]. Considering this together with our current observation, it may be inferred that a knee OA patient is more likely to undergo JSW narrowing when he or she has severer symptoms for a prolonged period. Conversely, if a patient has symptoms but they improve with time, the narrowing of JSW is less likely to progress. Such a relationship between the symptoms and the progression of JSW narrowing could be helpful for those attempting to understand OA pathology, because it may indicate that cartilage degeneration and the appearance of knee pain could be caused by the same, or closely linked mechanism(s). This result also implies that a therapy to inhibit that mechanism(s), if established, could be effective in both the reduction of symptoms (pain) and prevention of cartilage loss. Elucidation of such a mechanism(s) might be a key to developing a new but effective treatment for knee OA.

Restriction in joint motion is one of the clinical features of knee OA, which is closely associated with the disability of the subjects [40-42]. Current investigation has shown that the change of ROM could be related to the radiographic progression of the disease. Although ROM improved in the knees that escaped radiographic progression (JSW narrowing or osteophyte growth), such improvement was not observed with the knees that underwent the progression (Figure 6). This association between the change of ROM and radiographic changes tended to be more apparent with osteophyte growth than with the progression of JSW narrowing. Despite its significance, the time course of the change of ROM in OA knees is not known well. Clearly, ROM declines with the progression of the disease [16,43]. However, since restriction in ROM is partly caused by pain and swelling of the joint [44,45], ROM could be improved when these symptoms are alleviated. This scenario might explain the improvement of ROM observed here with the knees that evaded radiographic progression.

Although this study has revealed several novel aspects in the relationship between radiographic changes and clinical findings of knee OA, several cautions need to be exercised in understanding the results. First, it should be noted that the subjects of this study were patients with symptomatic knee OA. This could be a unique feature of the study in that it showed the prognosis of knee OA patients who visit clinics, but the results might be different from those in previous studies based on general populations that included knee OA subjects without apparent symptoms. Again, since all of the subjects had significant levels of symptoms at baseline, the change of symptoms in the study period could be smaller than that in other studies upon general populations. This might have hindered clarification of the relationship between radiographic changes and symptoms. Second, in the radiographic evaluation, the change of JSW in the patellofemoral compartment was not considered, though osteophyte growth in that compartment was evaluated. Third, in the physical examination, the presence of joint swelling was determined only by palpation, while it could be estimated more accurately by other methods such as magnetic resonance imaging (MRI) or ultrasonography. Fourth, although recent studies report the significance of subchondral bone lesions in the progression and appearance of symptoms with knee OA [46-48], such lesions were not evaluated. Despite these limitations, the results of this investigation are worth considering when attempting to treat this common, but tenacious disease.

## Conclusions

In the patients with medially involved knee OA, the progression of joint space narrowing was significantly correlated with the severity of symptoms. The symptoms tended to be worse for those with higher rates of narrowing, while no such correlation was found between the severity of symptoms and the growth of osteophytes. For knees that did not undergo radiographic progression, the ROM improved during the follow-up period, but the ROM improved little with knees that underwent JSW narrowing or osteophyte growth. These findings may be informative and worth consideration in the treatment of patients with knee OA.

## Abbreviations

AP: anteroposterior; AUC: area under the curve; BMI: body mass index; CRP: C-reactive protein; ESR: erythrocyte sedimentation rate; JKOM: the Japanese Knee Osteoarthritis Measure; JSW: joint space width; MRI: magnetic resonance imaging; NSAIDs: non-steroidal anti-inflammatory drugs; OA: osteoarthritis; PA: posteroanterior; ROC: receiver operating characteristics; ROM: range of motion; TGF-β1: transforming growth factor-β1; VAS: visual analogue scale.

## Competing interests

The authors declare that they have no competing interests.

## Authors' contributions

NF conceived the idea of the study. NF, KT, RM and YK recruited subjects and evaluated clinical findings. NF, KT and YK performed evaluation and measurement of radiographs. SY, SI, NT and SF were engaged in data entry and analyses. All authors read and approved the final manuscript.

## Pre-publication history

The pre-publication history for this paper can be accessed here:

http://www.biomedcentral.com/1471-2474/11/269/prepub
